# Tongue-tie Repair: Z-Plasty Vs Simple Release

**Published:** 2015-03

**Authors:** Jamshid Yousefi, Fariba Tabrizian Namini, Seyed Mohammad Ali Raisolsadat, Rowan Gillies, Azar Ashkezari, John G. Meara

**Affiliations:** 1 *Department of Pediatric Surgery, 22 Bahman Hospital, Islamic Azad University of Mashhad, Mashhad, Iran.*; 2 *Clinical Fellow, Program in Global Surgery and Social Change, Boston Children’s Hospital, Harvard Medical School, Boston, USA. *; 3 *Family Physician, 22 Bahman Hospital, Islamic Azad University of Mashhad, Mashhad, Iran.*; 4 *Department of Plastic and Oral Surgery, Boston Children's Hospital, Harvard Medical School, Boston, USA. *

**Keywords:** Management, Z-plasty, Tongue-tie

## Abstract

**Introduction:**

Ankyloglossia is a congenital anomaly in which the lingual frenulum is unusually short and thick, thus decreasing tongue mobility. In the context of the newborn or young infant it is a subject of ongoing controversy within and between medical specialties. The controversy involves not only the definition but also the management of this anomaly. A tight lingual frenulum is considered a minor malformation by some investigators. Usual treatments for ankyloglossia include speech therapy, as well as simple frenulotomy and frenuloplasty. The aim of this study was to compare the latter two methods with respect to postoperative results and complications.

**Materials and Methods:**

A total of 50 patients referred for surgical care were randomly assigned into two groups: simple release (frenulotomy ) or Z-plasty (frenuloplasty), and underwent a pre-surgical assessment. After 3 months, patients were followed with a scheduled interview and questionnaire comparing the outcomes of the two methods. The data were analyzed using SPSS version 18.

**Results:**

Surgery had a significant effect on all variables measured in our study (P<0.05). Z-plasty had a greater effect on articulation, breast pain, tongue movement and parent satisfaction than simple release (P<0.05). Z-plasty and simple release had the same effect on breast feeding, latching, and sucking.

**Conclusion:**

Z-plasty is the preferred surgical method to address tongue-tie due to a greater improvement in mother’s breast pain, pronunciation and speech, tongue movement, and parental satisfaction.

## Introduction

Tongue-tie or ankyloglossia is defined as ‘a congenital condition in which lingual movement is decreased and the tip of the tongue cannot be protruded beyond the lower incisor teeth because of a short and thick lingual frenulum’ (1,2). 

Approximately 3–4% (range, 0.02–10.7%) of infants are born with some degree of ankyloglossia (3-5). 

Tongue-tie is reported to be more prevalent in males than females, with a ratio of 2.6:1.0 (2,6). Although its existence has been recognized for centuries, the clinical significance along with the diagnosis and management of ankyloglossia in the pediatric population have long been controversial topics (6).

Ankyloglossia predominantly appears as a sole anomaly, although it is sometimes accompanied by other congenital anomalies such as cleft palate (7). As it has a hereditary nature and occurs more commonly in male children, it has been suggested to be related to the X-chromosome (7-9).

There have been numerous attempts to define tongue-tie. These include grading and scoring the degree of shortening of the frenulum (mild, moderate and severe) as well as noting thickness (thin or thick frenulum) and notching of the tongue, or functional assessments. Despite these attempts, a clear definition and severity classification have not yet been established (7,10).

Indications for surgery are variable, including breast feeding difficulties and speech problems depending on the patient’s age. Others include dental, social and mechanical problems. Survey results have demonstrated significant differences within and among the professional groups, with pediatricians being the least likely to recommend surgery. For example, Wright concluded from a retrospective study that there is no place for neonatal frenulotomy whereas Sanchez-Ruiz et al. reported problems with deglutition and dentition in older children with uncorrected lingual frenula (10,11). Feeding difficulties have been reported in 25–44% of infants with tongue-ties (4,12), leading to advocacy for the use of division of tongue-tie to improve breast-feeding in conjunction with calls for further research (12).

The difficulties in breast-feeding attributed to tongue-tie include difficulty in latching and maintaining latch, an inefficient feeding cycle, maternal pain or the sensation of chomping, and reduced milk supply (12,13).

## Materials and Methods

In a prospective clinical trial conducted between June 2009 and June 2011, we performed tongue-tie release using frenulotomy (simple release without suturing) or frenuloplasty (Z-plasty with opposing 60° triangular flaps alternated and sutured) in children under the age of 12 years. The population was drawn from those attending pediatric and pediatric surgery clinics in our teaching hospital in Mashhad (Northeastern Iran).

 The study was undertaken following the review and permission of the research affairs department of the medical school. All parents signed a consent form agreeing to be part of the study. Exclusion criteria were any congenital abnormality in the craniofacial region, abnormal mental development, any difficulty in feeding except that attributable to tongue-tie, and Hazelbaker’s appearance score >8 (18, 10). According to the inclusion criteria, we recruited 50 cases to the study, alternating inclusion into the two groups, with 25 children in each arm of the study. Parents were blinded to the allocated arm.

 The simple-release (frenulotomy ) group included six females and 19 males, while the Z-plasty group included seven female and 18 males. There was no statistically significant difference in terms of gender between the two groups. The total male-to-female ratio was 2.8 to 1.

The age of patients ranged from 5 days to a maximum of 8 years at time of surgery (mean, 32 months). Forty-eight percent of patients were diagnosed at an age of more than 1 year, 42% were diagnosed between 1 month and 1 year, while 10% were diagnosed in the neonatal period.

**Fig1:**
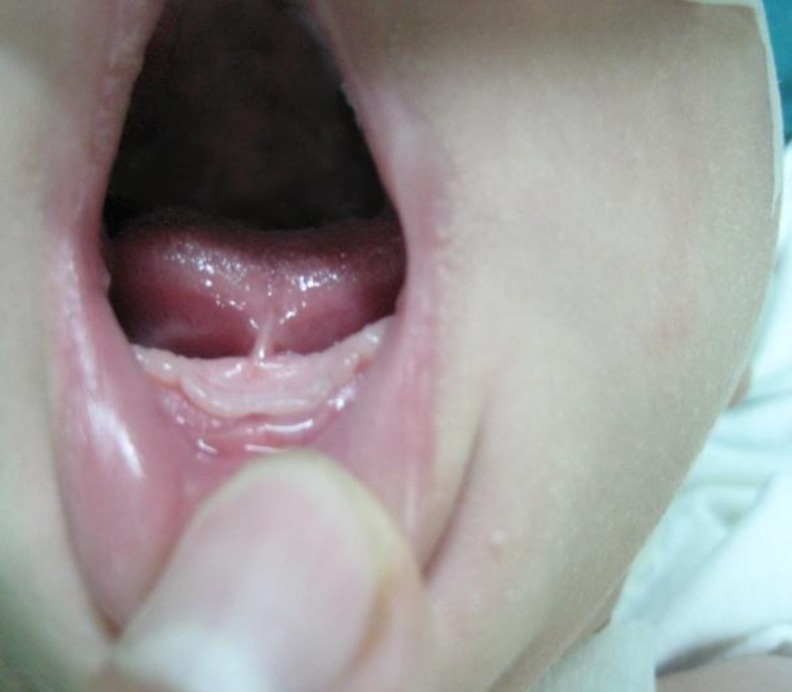
Tongue-tie diagnosed for mastalgia by a physician.

**Fig 2:**
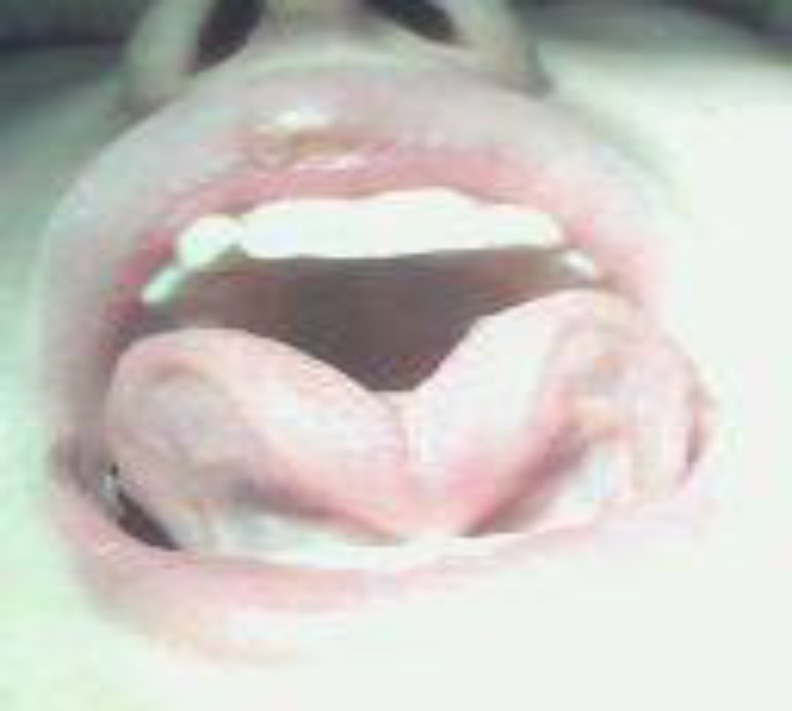
Tongue-tie diagnosed by the family due to misarticulation

The method of diagnosis was most commonly initiated by the mother (72% in the Z-plasty group and 80% in the simple-release group; Fig. 1), while the remaining patients were diagnosed by physicians (Fig. 2).

Hazelbaker’s appearance items were used to score severity because of the difficulty of functional scoring for our patients, especially small infants (10). The cut-off to enter the study was an appearance score <8. The mean score of patients was 6 and 6.20, respectively, for the Z-plasty and simple-release groups.

As shown in Table 1, there was no statistically significant difference between the two groups in Hazelbaker appearance score at baseline.

**Table 1:** Hazelbaker appearance score at baseline

**Hazelbaker score**		**Frequency**	**Mean**	**SD**	**P-value**
Z-plasty	≤ 5	11	6	0.20	0.60
	6	4			
	7	5			
	8	5			
Simple release	≤ 5	6	6.20	0.24	
	6	8			
	7	7			
	8	4			

Patients were assigned to two arms using an alternating ABBA system (A for Z-plasty and B for simple release). All procedures were performed under general anesthesia by the same pediatric surgeon. Before and immediately after the procedure, the frenulum and its new length were measured. There were no intraoperative complications. Post operatively, there was one case of minor hemorrhage not requiring surgical intervention in the Z-plasty group and one case of adhesion that required reoperation in the simple-release group. All patients were followed up after 3 months in a scheduled program. We controlled for the effect of surgery and compared the effects of the two methods in relation to breast feeding, jaw locking, tongue lengthening, word articulation, tongue movement, and parent satisfaction.

Eighteen cases in the Z-plasty group and nine cases in the simple-release group were of breast-feeding age. Eight (44%) and seven (77%) mothers, respectively, of these children reported milking pain. The numerical rate pain scoring (NRP) before and after surgery was used to calculate changes in breast pain.

Among patients who experienced articulation problems and/or could cooperate with functional scoring (12 cases in the Z-plasty group and 19 in the simple-release group), we evaluated the effect of the procedure on their articulation and tongue movements prior to surgery and after 3 months of follow-up. The mean Hazelbaker’s functional score before surgery was 9 in the Z-plasty group and 9.8 in the simple-release group.

We evaluated the effect of the two procedures on latching on in breast-feeding patients and we also measured the elongation of the tongue after surgery by measuring the frenulum before and after surgery. Finally we measured parental satisfaction after surgery using a subjective numerical scale. Collected data were analyzed using SPSS software, version 18. Paired t-tests and the Man-Whitney test were used for comparisons.

## Results

We evaluated the effect of surgery on tongue-tie and compared simple release (frenulotomy) with Z-plasty (frenuloplasty) by interviewing parents and children.

*Effect on breast feeding*

Twenty-seven patients were of breast-feeding age (18 in the Z-plasty group and nine in the simple-release group). Both methods had a statistically significant impact on improving breast feeding (Table. 2a).

**Table 2a:** Effect of surgery on breast feeding

**Procedure**	**Frequency**	**Mean**	**SD**	**t-Test**	**P-value**
Z-plasty	18	2.61	0523	22.084	0.000
Simple release	9	2.33	0.500	14.000	0.000

Scale; no change=0, improved=1, good improved=2, and full resolution of feeding problems=3

The Mann-Whitney test was used to compare the outcomes of these procedures for Z-plasty and simple release, and showed no difference between the methods (Table. 2b).

**Table 2b:** Effect of method of surgery on breast feeding

**Procedure**	**Frequency**	**Mean**	**Man-Whitney test**	**P-value**
Z-plasty	18	2.66	58.5	0.253
Simple release	9	2.33		

				

*Effect on mastalgia*

The result of surgery on breast pain is shown in Table 3a. Both methods had a statistically significant impact on mastalgia.

The Man-Whitney test showed no difference between the two methods in mastalgia (Table. 3b).

**Table 3a:** Effect of surgery on mastalgia

**Procedure**	**Frequency**	**Mean pain reduction**	**SD**	**t-Test**	**P-value**
Z-plasty	8	6.4	1.3	-0.14	0.000
Simple release	7	5.9	0.9	-0.17	0.000

**Table 3b:** Effect of method of surgery on mastalgia

**Procedure**	**Frequency**	**Mean**	**Man-Whitney Test**	**P-value**
Z-plasty	8	6.37		0.39
Simple release	7	5.71		
				
				

*Effect on articulation*

To assess articulation, we used a score based on words involving ‘D’, ‘T’ and’ N’, before and after surgery. Because of the complexity of speech development in every language, these words were chosen in consultation with a speech pathologist as they best assess the need to bring the tongue forward and upward as required in the Persian language. Table 4a shows the improvement in articulation, with Table 4b showing a greater improvement in the Z-plasty group.

**Table 4a:** Effect of surgery on word articulation

**Procedure**	**Frequency**	**Mean**	**SD**	**t-Test**	**P-value**
Z-plasty	12	3	0.603	17.234	0.000
Simple release	17	1.94	0.827	9.979	0.000
					

Scale; no change=1, good=2, better=3, best=4 three month after surgery

**Table 4b:** Effect of method of surgery on articulation

**Procedure**	**Frequency**	**Mean**	**Man- Whitney Test**	**P-value**
Z-plasty	12	3	39	0.001
Simple release	17	1.94		
				

*Effect on improvement of tongue movement*

Of our patients, 11 cases in the Z-plasty group and 20 in the simple-release group were able to perform the Hazelbaker’s functional scoring assessment. A significant improvement in tongue mobility for both methods is shown in Table 5a, while Table 5b shows the results of the Man-Whitney test, revealing an advantage for the Z-plasty technique.

We compared the effect of two procedures on tongue function, and found a greater improvement in mobility in the Z-plasty group than in the simple-release group.

**Table 5a:** Effect of surgery on tongue movement

**Procedure**	**frequency**	**mean**	**SD**	**t-Test**	**P-value**
Z-plasty	11	2.91	0.302	32.000	0.000
Simple release	20	2.10	0.553	16.998	0.000

**Table 5b:** Effect of method of surgery on tongue movement

**Procedure**	**Frequency**	**Mean**	**Man-Whitney**	**P-value**
Z-plasty	11	2.91	31.00	0.001
Simple release	20	2.10		

*Jaw locking (better latching)*

Table 6a shows a significant effect of both methods on jaw locking (better sucking) in breast-feeding children. 

There was no difference between Z-plasty and simple release in effect on jaw locking (Table.6b).

**Table 6a:** Effect of surgery on jaw locking

Procedure	**Frequency**	**Mean**	**SD**	**t-Test**	**P-value**
Z-plasty	19	2.79	0.631	19.282	0.000
Simple release	8	2.63	0.518	14.346	0.000
					
					

Latching score; weak=1, moderate=2, strong=3 for mothers.

**Table 6b:** Effect of two methods on jaw locking

**Procedure**	**Frequency**	**Mean**	**Man-Whitney Test**	**P-value**
Z-plasty	19	2.79	66.5	0.621
Simple Release	8	2.63		
				
				

*Tongue release and elongation*

There was a significant difference in both groups in the tip–base distance before and after release after 3 months (from 6.8 mm to 17.56 mm in the Z-plasty group and from 6.93 mm to 10.44 mm in the simple-release group)( Table.7).

**Table 7:** Effect of methods on tongue elongation

**Procedure**	**Frequency**	**Mean**	**SD**	**Man-Whitney Test**	**P-value**
Z-plasty	25	17.56	4.484	104.5	0.000
Simple release	25	10.44	3.787		
					

*Parent satisfaction*

A score of 1 to 10 was used for parental satisfaction. Table 8 shows a mean average satisfaction with Z-plasty of 10 (maximum gain) compared with 8.6 in the simple-release group. Thus, Z-plasty has a significantly greater effect on parent satisfaction (P<0.005).

**Table 8:** Parental satisfaction in the two groups

**Procedure**	**Frequency**	**Mean**	**SD**	**Man-Whitney Test**	**P-value**
Z-plasty	25	10	0.000	225.0	0.005
Simple release	25	8.6	2.291		

## Discussion

Ankyloglossia or tongue-tie is a known and common entity, but there is no clear agreement as to its management. Some authors disagree with surgery and some advocate for surgery. In a survey by Mansser (3) on feeding, speech, and social/mechanical problems in these patients, different specialties had different opinions. Surgery is recommended at least sometimes for feeding, speech, and social/mechanical issues by 53, 74, and 69% of otolaryngologists, respectively, but by only 21%, 29%, and 19% of pediatricians.

In a survey conducted in Australia by Brinkmann (6), almost three-quarters of surgeons who responded said they practiced surgical release of ankyloglossia. This survey also identified a strong belief that surgical intervention was necessary; only 10% of respondents believed that it was rarely indicated. This belief by Australian surgeons is in contrast with the opinion of the Community Pediatrics Committee of the Canadian Pediatric Society (14), which opines that most of the time tongue-tie is an anatomical finding without significant consequences for breast-feeding. This body suggests that surgical intervention is not usually warranted, but may be necessary if significant tongue-tie is associated with major breast-feeding problems.

Despite the debate, the releasing of tongue-tie is a common procedure that is performed by many surgeons, nurse practitioners and also midwifes (15).

The indication for surgery varies, but the two most common indications are feeding problems and speech/articulation problems (1,10,16). Other indications are dental and social problems.

The time of intervention also varies, with the neonatal period being the most common, related to feeding problems. Intervention can also be recommended for dental reasons in the first year of life (17). Because it is not routine to check for the presence of tongue-tie in the neonate, most of our patients were diagnosed by their parents across a range of ages. On review of the literature, we were not able to find reports on the mode of diagnosis, so our observation appears to be a new finding.

Tongue-tie is most common in boys, with the reported male-to-female ratio ranging from 1.7:1 (10) to 2.6:1 (1), which is close to our result (2.8 to 1).

The age of diagnosis is different in various reports, ranging from 3.2% in the first and second days of life to 12.8% in outpatients (10). The incidence in Iran is unknown; however it is interesting to note that 30% of our patients of breast-feeding age presented with feeding problems. This is a much greater proportion than that previously reported by Ballard and Hazelbaker (10,18).

One of the most controversial aspects of the management of tongue-tie is the evaluation and scoring of the severity of disease. It appears that the Hazelbaker’s appearance and functional scoring is the most reliable (19), but it is unclear how the function of the tongue of a neonate can be evaluated. For this reason, we used only the appearance items of Hazelbaker’s scoring as an indication for surgery for neonates (Table. 1). Another controversial matter is the method of releasing of tongue-tie. In Middle Ages, this was done using the finger nail of midwifes (20); however, more recently, Z-plasty with genioglossus muscle release (21), yttrium aluminum garnet (YAG) laser (22), and conventional frenulotomy and frenuloplasty have been widely adopted.

A Medline literature search revealed no papers describing a comparison of methods or recommendations for which should be preferred in different circumstances. Most articles describe simple release with scissors with or without general anesthesia; however reoperation rates are as high as one-third (16), with some reports of releasing and suturing (frenuloplasty), or performing flaps (Z-plasty) in these cases (21). A further area of conflict is whether frenuloplasty (releasing and suturing) should be performed with or without anesthesia (16). The procedure can be performed with or without local anesthesia or under general anesthesia (16,23) as in all of our cases.

In contrast to many studies, the most common indication for our patients was speech/articulation (58 %), similar to the result found by Klockar (16), and the second was breast feeding (54%), mostly determined by the patient’s age. The majority of mothers were prompted to bring their children based on the appearance of the tongue-tie, not because of feeding or any breast pain. Feeding difficulties and breast pain were only elucidated on history and examination.

In general, our patients had an excellent response to the tongue-tie release (simple release or Z-plasty), consistent with other reports (5,10,12,24,25). Breast-feeding improvement was similar for both methods (Table. 2b).

In terms of speech/articulation, we report good results from the release of tongue-tie, as in other studies (26,16). In a study by Klockars in which the dominant indication was speech problems (64%) an 84% rate of parental satisfaction was reported (16). This was similar to our result in the simple-release group; while with Z-plasty we had a maximal result (100%; Table. 8). We also found that the Z-plasty has a better effect on articulation than simple release (Table. 4b). Perhaps related to this, we also found an improved result for Z-plasty in both tongue movements and Hazelbaker’s functional items (Table. 5).

We consider these findings to be novel, as there are no studies in the English literature comparing these two techniques.

## Conclusion

Although tongue-tie releasing is a common surgery for breast feeding, speech, dental, and social problems, there is no general consensus regarding the indication and method of surgery. In this study we showed that the release of tongue-tie has a significant positive effect on these problems and that Z-plasty has better impact on speech/articulation, tongue movement and parent satisfaction. These findings are new and should be examined in a larger study to validate these conclusions. 

## References

[1] Messner AH, Lalakea ML, Aby J, Macmahon J, Bair E (2000). Ankyloglossia: incidence and associated feeding difficulties. Arch Otolaryngol Head Neck Surg.

[2] Segal LM, Stephenson R, Dawes M, Feldman P (2007). Prevalence, diagnosis, and treatment of ankyloglossia: methodologic review. Can Fam Physician.

[3] Messner AH, Lalakea ML (2000). Ankyloglossia: controversies in management. Int J Pediatr Otorhinolaryngol.

[4] Griffiths M, Westcott C, Hogan M (2005). A randomized controlled trial of division of tongue-tie in infants with feeding problems. Journal of Paediatrics and Child Health.

[5] Edmunds J, Miles SC, Fulbrook P (2011). Tongue-tie and breastfeeding: a review of the literature. Breastfeed Rev.

[6] Brinkmann S, Reilly S, Meara JG (2004). Management of tongue-tie in children: A survey of pediatric surgeons in Australia. Journal of Paediatrics and Child Health.

[7] Han SH, Kim MC, Choi YS, Lim JS, Han KT (2012). A Study on the Genetic Inheritance of Ankyloglossia Based on Pedigree Analysis. Arch Plast Surg.

[8] Pauws E, Moore GE, Stanier P (2009). A functional haplotype variant in the TBX22 promoter is associated with cleft palate and ankyloglossia. J Med Genet.

[9] Morowati S, Yasini M, Ranjbar R, Peivandi AA, Ghadami M (2010). Familial ankyloglossia (tongue-tie): a case report. Acta Med Iran.

[10] Ballard JL, Auer CE, Khoury JC (2002). Ankyloglossia: assessment, incidence, and effect of frenuloplasty on the breastfeeding dyad. Pediatrics.

[11] Wright JE (1995). Tongue-tie. Journal of Paediatrics and Child Health.

[12] Berry J, Griffiths M, Westcott C (2012). A Double-Blind, Randomized, Controlled Trial of Tongue-Tie Division and Its Immediate Effect on Breastfeeding.

[13] Fitz-Desorgher R (2003). All tied up-tongue tie and its implications for breastfeeding. Pract Midwife.

[14] Community Pediatrics Committee (2002). Canadian Pediatric Society position statements: ankyloglossia and breastfeeding. J Paediatr Child Health.

[15] Amir LH, James JP, Kelso G, Moorhead AM (2011). Accreditation of midwife lactation consultants to perform infant tongue-tie release. Int J Nurs Pract.

[16] Klockars T, Pitkäranta A (2009). Pediatric tongue-tie division: indications, techniques and patient satisfaction. Int J Pediatr Otorhinolaryngol.

[17] Kupietzky A, Botzer E (2005). Ankyloglossia in the infant and young child: clinical suggestions for diagnosis and management. Pediatr Dent.

[18] Hazelbaker AK (1993). The Assessment Tool for Lingual Frenulum Function (ATLFF): Use in a Lactation Consultant Private Practice.

[19] Amir LH, James JP, Donath SM (2006). Reliability of the Hazelbaker Assessment Tool for Lingual Frenulum Function. Int Breastfeed J.

[20] Obladen M (2010). Much ado about nothing: two millennia of controversy on tongue-tie. Neonatology.

[21] Choi YS, Lim JS, Han KT, Lee WS, Kim MC (2011). Ankyloglossia correction: Z-plasty combined with genioglossus myotomy. J Craniofac Surg.

[22] Kotlow L (2011). Diagnosis and treatment of ankyloglossia and tied maxillary fraenum in infants using Er: YAG and 1064 diode lasers. Eur Arch Paediatr Dent.

[23] Glynn RW, Colreavy M, Rowley H, Gendy S (2012). Division of tongue tie: Review of practice through a tertiary paediatric otorhinolaryngology service. Int J Pediatr Otorhinolaryngol..

[24] Miranda BH, Milroy CJ (2010). A quick snip - A study of the impact of outpatient tongue tie release on neonatal growth and breastfeeding. J Plast Reconstr Aesthet Surg.

[25] Kumar M, Kalke E (2012). Tongue-tie, breastfeeding difficulties and the role of Frenotomy. Acta Paediatr.

[26] Messner AH, Lalakea ML (2002). The effect of ankyloglossia on speech in children. Otolaryngol Head Neck Surg.

